# Tubeless mini-percutaneous nephrolithotomy to remove a staghorn stone concurrent with proximal ureteral calculus in an elderly patient: A case report

**DOI:** 10.1016/j.eucr.2023.102332

**Published:** 2023-01-23

**Authors:** Thanathorn Thampravit, Yada Phengsalae, Chinnakhet Ketsuwan

**Affiliations:** Division of Urology, Department of Surgery, Faculty of Medicine Ramathibodi Hospital, Mahidol University, Bangkok, Thailand

**Keywords:** Elderly, Mini-PCNL, Staghorn calculus

## Abstract

A complex staghorn calculus that is concurrent with an impacted large proximal ureteric calculi is rarely found in elderly patients, and morbidity and mortality rates are much higher if left untreated. We report the case of an 88-year-old female with complex high-volume renal and ureteral calculus who was treated successfully using a tubeless mini-percutaneous nephrolithotomy. The entire stone was retrieved, and the patient fully recovered without any complications.

## 1 Introduction

In well-developed countries, a staghorn calculus that is concurrent with an impacted large proximal ureteric calculi rarely presents with potentially diminished kidney function of the affected side. The morbidity and mortality rates are much higher if left untreated, since significant complications from urinary tract infections and obstructions may contribute to a life-threatening event. Nonetheless, conservative care for active treatment should not be offered as an option to healthy patients, because it also carries a large mortality rate. Therefore, staghorn calculus is a serious disease entity that should be managed with an effective and aggressive therapeutic modality. Surgical treatment for stone disease, including extracorporeal shock wave lithotripsy, uretero-renoscopic lithotripsy, and percutaneous nephrolithotomy (PCNL), has undergone dramatic changes since the establishment of minimally invasive technology.^1^^,^^2^ Miniature percutaneous nephrolithotomy (mini-PCNL) has been introduced by miniaturization of the equipment and improvement of PCNL systems with the aim of a high stone clearance rate comparable to the standard PCNL and potentially decreased complication and pain rates afforded by the smaller tract size.

To the best of our knowledge, this is the first clinical case reporting tubeless mini-PCNL in an elderly patient with a staghorn stone concurrent with a large ureteric calculus.

## 2 Case report

**Fig. 1:**
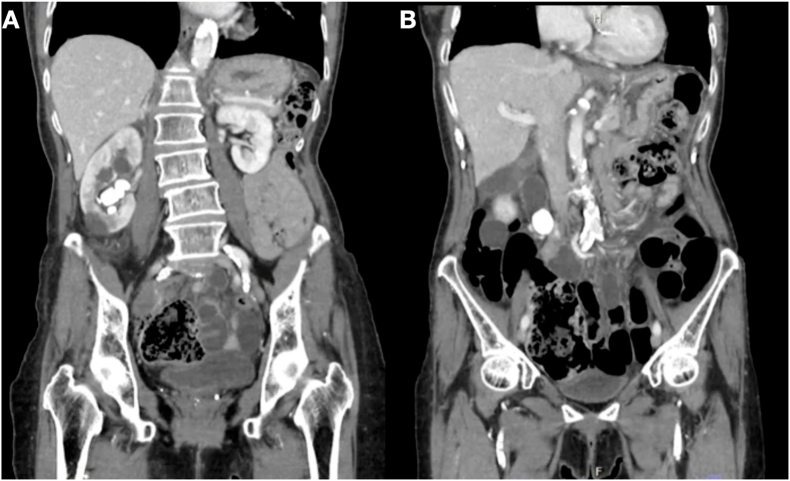
A, Computerized tomography scan showing a complex staghorn stone; B, proximal ureteric stone.

An 88-year-old female patient was sent to our clinic because of intermittent right renal colic. Her medical problems included hypertension, dyslipidemia, primary hypothyroidism, and bronchiectasis. A meticulous preoperative evaluation and risk assessment by an anesthesiologist revealed that the patient's overall physical health was still suitable for the surgery. Urinalysis demonstrated modest pyuria, but negative urine cultures. The serum creatinine level was 1.2 mg/dL. A preoperative computed tomography scan illustrated a staghorn calculus 4.5 cm in size (Fig. 1A) concurrent with a proximal ureteric stone 2.5 cm in size (Fig. 1B). Excretion was mildly decreased in the right kidney, but normal excretion was observed on the left side. After discussing the various treatment options with the patient, we concluded that mini-PCNL in the prone position would be the preferred choice.

**Fig. 2:**
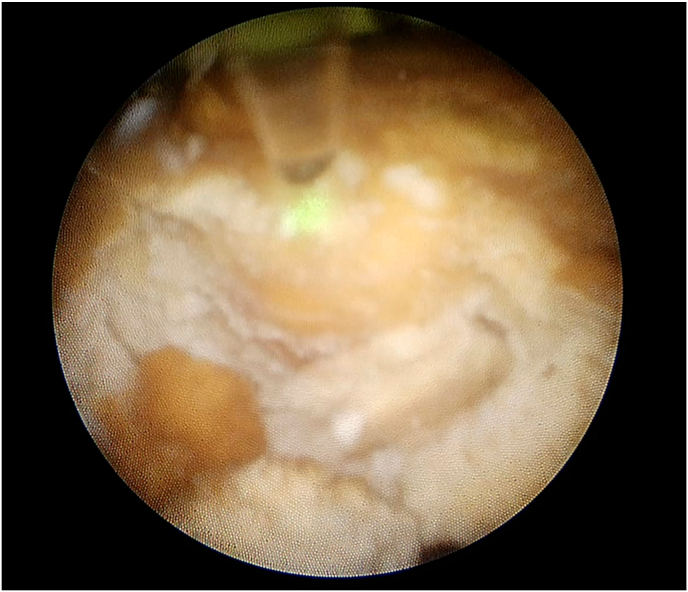
Miniature nephoscope view of the kidney stone.

**Fig. 3:**
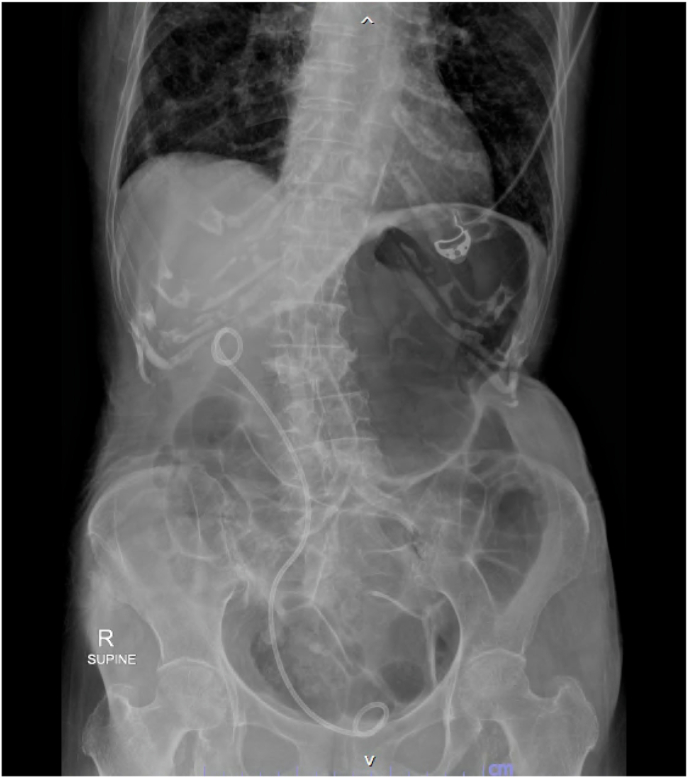
Post-operative abdominal plain radiography showing no evidence of residual stones.

Intravenous prophylactic antibiotics (cefuroxime) were administered during the induction phase of general anesthesia. The patient was arranged into the lithotomy position. A 6F ureteral catheter was inserted cystoscopically, passed into the renal pelvis of the right side, and illustrated the collecting system with contrast dye. We then turned the patient to a prone position. An access needle (18 gauge, 20 cm) was punctured into the upper-pole calyx under fluoroscopic guidance. Tract dilatation was achieved using a one-shot dilator over the guidewire to create a 15F working channel. Laser lithotripsy was performed using a 120 W Ho:YAG system (Lumenis, San Jose, CA) and a 550 μm core laser fiber with the fragmentation technique (Fig. 2), which employs energy settings of 1.5 J and a rate of 30 Hz. The stone fragments were retrieved with grasper forceps or Venturi effect through a 12 Fr nephroscope MIP-M system (Karl Storz, City, Germany). At the end of the procedure, an antetrograde 8Fr double-J stent was placed. Total operative time was 90 minutes and laser time was 28 minutes. We obtained plain films of the abdomen to assess residual stones and confirmed that the patient kept the double-J stent in the appropriate position (Fig. 3). No residual stones were evident. The patient had an uneventful postoperative recovery. The stent was removed cystoscopically 6 weeks after discharge. The stone composition was not evaluated due to financial constraints.

## 3 Discussion

Globally, life expectancy has steadily increased for nearly 200 years. Healthy longevity has been recognized in many countries, including the United States, other developed countries, and Thailand. Nephrolithiasis is one of the most challenging disorders for clinicians, and strategies for disease management require both medical and surgical interventions.^3^ PCNL is the treatment of choice for staghorn and large-volume complex renal calculi, as well as upper tract calculi that have failed other modalities. Generally, PCNL is a safe procedure that is well tolerated, but as with any surgical intervention, various severe adverse associated events can still occur.

In the last two decades, not only have endoscope and lithotripsy innovations improved, but the techniques of PCNL have also been adjusted. This progression makes mini-PCNL a valuable option for retrieving both large and progressively smaller stone sizes. PCNL using small instruments and a small caliber working sheath, called mini-PCNL or mini-perc, was first presented for the treatment of pediatric renal stones in 1997. Mini-PCNL is defined as a sheath size less than 20Fr–24Fr, whereas standard PCNL utilizes a sheath size of 24Fr–30F; however, an exact definition does not exist now. Furthermore, mini-PCNL is subclassified as ultra-mini and micro at 11Fr–13Fr and less than 10Fr, respectively.^4^^,^^5^ Mini PCNL represents similar stone-free rates with lower complications compared with standard PCNL. A smaller sheath size leads to less renal trauma, bleeding, and the need for transfusion. Moreover, a special phenomenon, the Venturi effect, referred to as the “vacuum cleaner effect,” takes benefits from the fluid dynamic property to produce a low-pressure eddy 5 mm from the tip of the lens to pull out stone fragments quickly when the scope is withdrawn. The tubeless option was appropriate in this case, as it helps reduce postoperative pain, analgesia requirements, and hospital stays. We confirmed the excellent results of the mini-PCNL procedure by the absence of any pieces of residual stone in a plain film of the abdomen administered postsurgery.

## 4 Conclusion

We successfully treated complex renal calculi and proximal ureteral calculi using miniature percutaneous nephrolithotomy. This procedure appears to provide a valuable option for an elderly patient who needs complete stone clearance within a single procedure.

## Declaration of competing interest

None.
